# A Comparative Study of Signal Representations Methods and Deep Learning Architectures for PPG-Based Cuffless Blood Pressure Estimation

**DOI:** 10.3390/s26092847

**Published:** 2026-05-02

**Authors:** Han Zhang, Xudong Hu, Xizhuang Zhang, Zhencheng Chen, Yongbo Liang, Gang Wang

**Affiliations:** 1 School of Electronic Science and Engineering, University of Electronic Science and Technology of China, Chengdu 611731, China; 2 School of Life and Environmental Sciences, Guilin University of Electronic Technology, Guilin 541004, China; 3 Ningbo Ming Sing Optical R&D Co., Ltd., Ningbo 315104, China; 4 Guangxi Key Laboratory of Metabolic Reprogramming and Intelligent Medical Engineering for Chronic Diseases, Guilin 541004, China; 5 The Key Laboratory of Biomedical Information Engineering of Ministry of Education, Institute of Health and Rehabilitation Science, School of Life Science and Technology, Xi’an Jiaotong University, Xi’an 710049, China

**Keywords:** photoplethysmography (PPG), blood pressure prediction, intelligent medicine, deep learning

## Abstract

Hypertension is a major risk factor for cardiovascular disease and requires effective long-term monitoring. Photoplethysmography (PPG), acquired from wearable optical sensors, offers a convenient and non-invasive signal source for cuffless blood pressure (BP) estimation, but existing studies have mainly emphasized model architecture optimization, with limited systematic investigation of signal representation. This study systematically compares seven one-dimensional-to-two-dimensional signal transformation methods and evaluates multiple architectural variants for PPG-based cuffless BP estimation under a unified framework. Experiments were conducted using PPG and arterial BP signals from the UCI Open Blood Pressure Database. The best-performing configuration, based on continuous wavelet transform (CWT), achieved estimation errors of 3.80 ± 5.02 mmHg for systolic BP and 1.65 ± 2.70 mmHg for diastolic BP. Further real-world validation on 26 participants using an Omron cuff-based monitor as the reference showed good consistency, with correlation coefficients of R = 0.96 for SBP and R = 0.74 for DBP. The results demonstrate that appropriate signal representation, particularly CWT, plays a critical role in improving estimation accuracy and robustness, and may facilitate the development of wearable cuffless BP monitoring systems.

## 1. Introduction

Cardiovascular disease (CVD) has emerged as a critical global public health challenge, with hypertension being a major contributor. Recent data indicate that hypertension affects approximately 26% of adults worldwide, with a particularly high prevalence in China of 27.9% [1]. This prevalence is rising as the population ages, emphasizing the urgent need for effective prevention and treatment.

Hypertension typically manifests with an insidious onset and is often asymptomatic during its early phases, which frequently results in delayed clinical intervention. If left uncontrolled, hypertension can precipitate severe cardiovascular sequelae, encompassing ischemic stroke, myocardial infarction, and chronic kidney disease [2]. Current clinical BP management relies on periodic measurement and medication adjustment, but traditional measurement methods have inherent limitations. Invasive arterial puncture, while the gold standard for accuracy, is only applicable to critically ill patients in clinical settings. Non-invasive cuff-based methods, such as the auscultatory and oscillometric techniques, are widely used for their simplicity but fail to support continuous monitoring and are ill-suited for wearable sensor applications due to cuff-related discomfort and movement artifacts [2].

Photoplethysmography (PPG) has emerged as a highly promising candidate for non-invasive, continuous physiological monitoring, offering advantages of low cost, ease of integration, and non-obtrusive data acquisition [3]. PPG sensors detects subtle changes in vascular blood volume via optoelectronic sensing, and its close correlation with hemodynamic changes makes it a viable signal source for cuffless BP prediction. With the advancement of deep learning, an increasing number of studies have explored PPG-based cuffless BP prediction, leveraging the ability of deep neural networks to automatically extract complex, non-linear features from physiological signals—an advantage over traditional manual feature extraction methods. For instance, Baek et al. [4] proposed an end-to-end fully convolutional network for direct BP prediction from raw PPG signals, eliminating the need for handcrafted features. Wu et al. [5] showed that continuous wavelet transform (CWT) representations can improve BP classification performance from PPG when combined with deep learning. Wang et al. [6] combined visibility graph-based feature extraction with transfer learning to enhance prediction accuracy. Al Fahoum et al. [7] further developed a CWT-scalogram-based PPG framework with a dedicated PPG-NET architecture for hypertension classification.

Despite these advancements, several important challenges remain in PPG-based cuffless BP estimation. Most existing studies focus on iterative optimization of deep learning architectures, whereas the effect of signal representation on estimation performance has received relatively limited systematic investigation [8]. The choice of signal representation determines how BP-related morphological and time–frequency information is preserved and presented to the model, and an unsuitable representation may limit the achievable performance. In addition, many high-performing models remain insufficiently validated in real-world scenarios, creating a gap between laboratory performance and practical applicability [2,8]. This concern is further supported by the recent large-scale analysis of paired PPG–cuff BP data by Solà et al. [9], which highlighted both the potential of PPG for digital health applications and the continuing difficulty of identifying robust BP-related markers that generalize across populations and measurement conditions.

**Figure 1:**
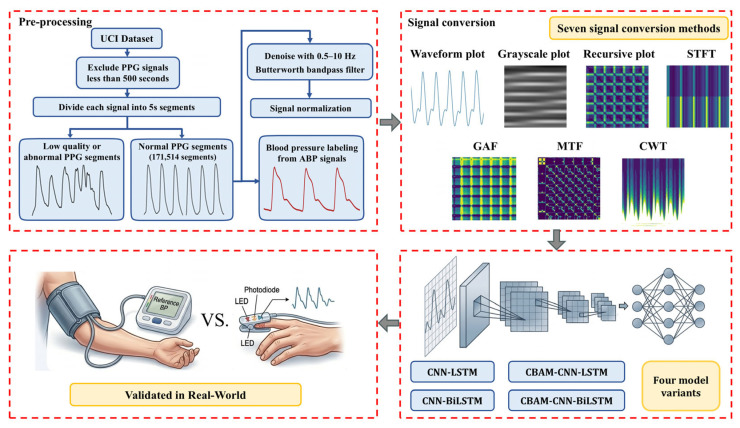
The workflow of this study. Note: PPG and ABP signal refer to photoplethysmography and arterial blood.

To address these issues, this study systematically compares seven signal transformation methods and multiple architectural variants for PPG-based blood pressure estimation. The overall workflow of the study is shown in Figure 1. The primary contributions of this work are summarized below:

1. A unified experimental framework was designed to systematically compare seven commonly used PPG signal representation methods under identical preprocessing and prediction settings, enabling a controlled investigation of the role of input representation in cuffless BP estimation.
2. Several representative deep learning architectures were evaluated under the same input setting to assess the effects of attention mechanisms and temporal modeling, and to distinguish their contribution from that of signal representation.
3. The results show that signal representation exerts a larger influence on prediction performance than architectural refinement, with CWT demonstrating the most competitive performance among the evaluated methods.

## 2. Materials and Methods

### 2.1. Datasets

The dataset utilized in the present work is sourced from the University of California, Irvine (UCI) Machine Learning Repository, specifically the Cuff-Less Blood Pressure Estimation Data Set [10]. As a curated subset of the Medical Information Mart for Intensive Care (MIMIC) database, this collection was established to support multi-parameter intelligent monitoring [11].

The UCI dataset aims to extract high-quality data from the extensive MIMIC database. It comprises physiological data from 12,000 intensive care unit patients, including simultaneous recordings of the Electrocardiogram (ECG) signal, Photoplethysmogram (PPG) signal, and Arterial Blood Pressure (ABP) signal, all sampled at 125 Hz. The ECG signal is obtained from a chest wall patch electrocardiograph, the PPG signal from a fingertip photoplethysmograph, and the ABP signal from invasive blood pressure measurements.

**Figure 2:**
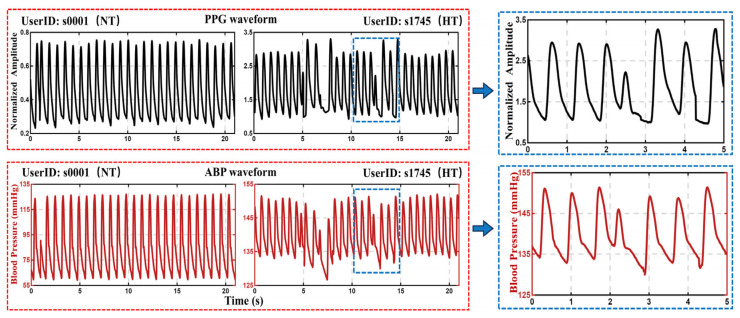
Schematic diagram of PPG and ABP signal data of a patient. Note: PPG and ABP refer to photoplethysmography and arterial blood pressure, respectively. NT and HT refer to Normotensive and Hypertension.

The UCI Open Blood Pressure dataset is widely recognized for its accessibility and utility. For our analysis, we exclusively utilized the PPG signal, with the ABP signal serving as the reference for blood pressure measurements. Figure 2 shows the PPG and ABP waveforms from two representative samples.

### 2.2. Signal Pre-Processing

To ensure the quality of our analysis, we performed several pre-processing steps on the biosignals from the UCI dataset:

(a) Data selection: Initially, we excluded data with a duration of less than 500 s, following the approach by Wang et al. (2021) [12]. This step resulted in a sample of 1741 subjects.
(b) Data segmentation: The remaining data were divided into segments using a 5 s window. This segmentation technique, supported by prior research [13], yielded a total of 191,598 segments.
(c) Reference-based filtering: Using reference blood pressure values obtained from corresponding ABP signals, we retained 190,114 signal segments. Additionally, we removed unreliable PPG signal segments based on abnormal blood pressure values or missing data were considered unreliable [14].

Signal quality assessment is crucial due to potential signal disturbances caused by various factors, including environmental interference (e.g., industrial frequency), and sensor placement errors. Such disturbances introduce noise into the collected signals, significantly impacting the accuracy of our analysis results [15,16]. Presently, signal quality assessment relies on factors such as frequency distribution, power spectrum distribution, and morphological distribution during signal processing [17].

**Figure 3:**
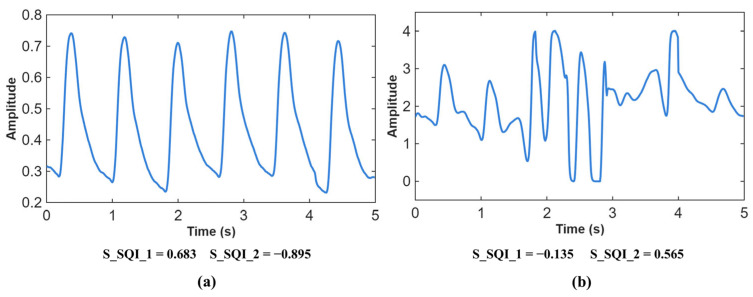
PPG signal segments are divided into two categories: (**a**) signal segments containing systolic and diastolic waveforms with repulsor cuts; (**b**) signal segments contaminated with noise and without clear systolic and diastolic waveforms. Note: S_SQI_1 and S_SQI_2 represent skewness and kurtosis of 5 s length PPG signal, respectively.

In this study, we employed two key metrics, skewness and kurtosis (as depicted in Figure 3). Each PPG signal segment was evaluated using a classification threshold, categorizing it as either an excellent or unsuitable PPG waveform. Segments with skewness greater than 0.1 and kurtosis less than −0.1 were retained [18,19]. This process resulted in the retention of 171,514 signal segments.

Following this assessment, we applied signal filtering to reduce noise inherent in sensor-acquired PPG signals. Given that the primary frequency range of pulse wave signals typically falls below 10 Hz, we employed a Butterworth filter with a range of 0.5–10 Hz [20]. Subsequently, the filtered PPG signals were normalized using mean-variance scaling to ensure uniform data scaling. Table 1 summarizes the sequential preprocessing steps applied to the raw biosignal data, including subject selection based on recording duration, signal segmentation using a fixed time window, and reference blood pressure filtering. The table also reports the number of subjects or signal segments retained after each step, providing a clear overview of data reduction throughout the preprocessing pipeline.

### 2.3. Signal Transformation Methods Under a Fixed Prediction Framework

To identify a more suitable input representation for blood pressure estimation, seven commonly used one-dimensional-to-two-dimensional signal transformation methods were systematically evaluated, including Waveform maps, Grayscale diagrams, Recurrence plots (RPs), Short-time Fourier transform (STFT), Gramian angular field (GAF), Markov transition field (MTF), and continuous wavelet transform (CWT). These methods differ in their ability to preserve the morphological, structural, and time–frequency characteristics of the original PPG signal [21]. Table 2 shows a comparison of the seven commonly used PPG signal conversion methods.

**Figure 4:**
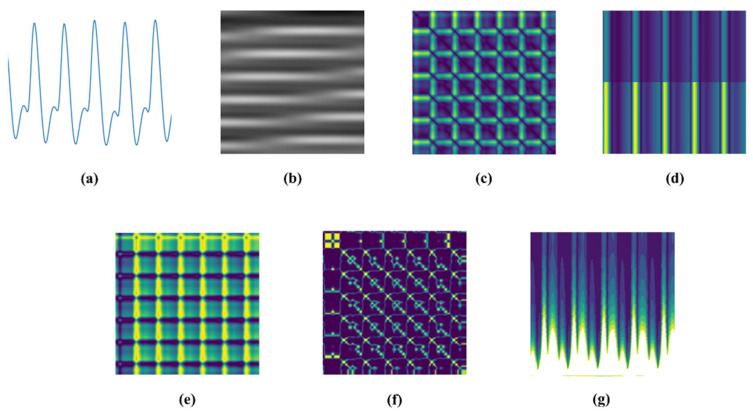
Seven different signal transformation methods. (**a**) Waveform maps; (**b**) Grayscale diagrams; (**c**) Recurrence plots; (**d**) Short-Time Fourier Transform; (**e**) Gramian Angular Field; (**f**) Markov Transition Field; (**g**) Continuous Wavelet Transform.

To ensure a fair comparison, all transformation methods were applied to the same preprocessed PPG segments, and the resulting images were evaluated using an identical downstream prediction framework. In this way, performance differences could be primarily attributed to the signal representation method itself rather than to variations in preprocessing or model architecture. Figure 4 illustrates various signal transformation methods.

### 2.4. Deep Learning Architectural Variants Under a Fixed Input Representation

To assess the effect of model architecture on blood pressure prediction, several network variants were compared under a fixed input representation. In contrast to Section 2.3, which evaluates different signal transformation methods using the same prediction framework, this section focuses on model-side variation while keeping the input unchanged.

**Figure 5:**
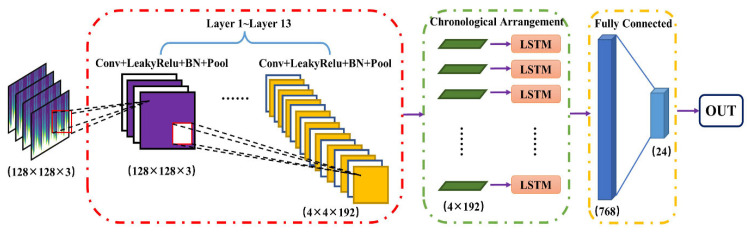
Network structure of CNN-LSTM model. Note: LSTM stands for Long Short-Term Memory; BN stands for Batch Normalization.

A CNN-LSTM network was adopted as the baseline model, where CNN was used to extract spatial features from transformed PPG images and LSTM was used to model sequential dependencies. The detailed architecture of the baseline model is listed in Table 3, and its overall structure is shown in Figure 5.

**Figure 6:**
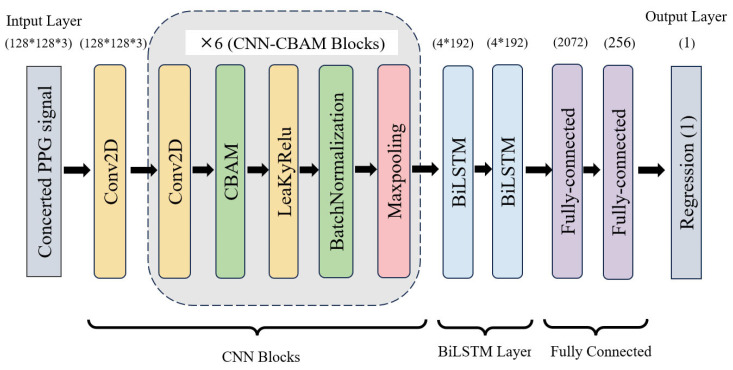
Network structure of CBAM-CNN-BiLSTM model. **Note:** CBAM stands for Convolutional Block Attention Module, CNN stands for Convolutional Neural Networks and BiLSTM stands for Bi-directional Long Short-Term Memory.

Based on the baseline model, two widely used architectural enhancement modules, CBAM [28] and BiLSTM, were introduced to refine feature representation and temporal modeling. Accordingly, three additional variants, namely CNN-BiLSTM, CBAM-CNN-LSTM, and CBAM-CNN-BiLSTM, were constructed for comparison. The composition of these architectural variants is summarized in Table 4, and the overall structure of the final CBAM-CNN-BiLSTM model is illustrated in Figure 6.

### 2.5. Evaluation Metrics

Before model training and performance evaluation, the evaluation metrics of the training model should be determined first. In this paper, the results are tested using Mean Error (*ME*), Mean Absolute Error (*MAE*), and Standard Deviation of Mean Absolute Error (STD). The mathematical formulations of these metrics are presented below:

(1) $$ME=\frac{1}{n}\sum_{i=1}^{n}\left(y_{\mathrm{pred}}^{\left(i\right)}-{y_{\mathrm{true}}}^{\left(i\right)}\right)$$

(2) $$MAE=\frac{1}{n}\sum_{i=1}^{n}\left|y_{\mathrm{pred}}^{\left(i\right)}-{y_{\mathrm{true}}}^{\left(i\right)}\right|$$

(3) $$STD=\sqrt{\frac{1}{n}\sum_{i=1}^{n}\left({y^{\left(i\right)}}_{\mathrm{pred}}-E\left({y^{\left(i\right)}}_{\mathrm{pred}}-{y_{\mathrm{true}}}^{\left(i\right)}\right)\right)}$$

where *n* represents the number of samples, *i* represents one of the samples, *y(i)* pred represents the predicted blood pressure, and *y(i)* true represents the true value of blood pressure.

### 2.6. Experimental Settings

This work incorporated 171,514 signal segments derived from 1741 participants. The full dataset was partitioned into training (80%, 137,211 segments) and test (20%, 34,303 segments) subsets via random splitting. Performance evaluation was conducted at the segment level, where each segment from a given subject was treated as an independent sample, and no subject-level aggregation was applied. Therefore, the current protocol represents a segment-level comparative evaluation rather than a strict subject-independent assessment.

All experimental implementations were conducted on a high-performance workstation configured with an Intel Xeon E5-2690 v4 CPU, four Nvidia Titan Xp GPUs (12 GB memory per device), and 128 GB of RAM. To mitigate overfitting during model training, data augmentation techniques were employed. We utilized the adaptive moment estimation (Adam) optimizer, with mean absolute error (MAE) serving as the optimization objective. The initial learning rate was initialized to $10^{-3}$, and a dynamic learning rate scheduling mechanism was implemented: if the MAE on the validation set did not improve for 100 consecutive epochs, the learning rate was adjusted by multiplying it by a factor of 0.96. The batch size was set to 256, and all models were trained for a maximum of 1000 epochs.

## 3. Results

### 3.1. Comparative Analysis of Signal Processing Methods

To evaluate the effect of input representation independently of model architecture, seven signal transformation methods were compared using the same CNN-LSTM framework. The corresponding prediction results are summarized in Table 5.

Among the seven methods, the two time–frequency-based approaches, STFT and CWT, outperformed the other five methods in both systolic and diastolic blood pressure estimation. Both methods achieved consistently lower MAE and STD compared to the other five approaches. Among all compared methods, CWT yielded the best overall performance. To better quantify the relative performance improvements, Table 6 presents the percentage gains in ME, MAE, and STD for each method compared to the baseline Waveform Maps approach. Positive values indicate better performance than the baseline, whereas negative values indicate degradation. The gains are calculated separately for systolic and diastolic blood pressure estimation.

### 3.2. Architectural Modifications of the Proposed Model

To evaluate the effect of model architecture independently of signal representation, four network variants were compared using the CWT input, which achieved the best performance in Section 3.1. The corresponding results are summarized in Table 7.

Compared with the baseline CNN-LSTM model, both CNN-BiLSTM and CBAM-CNN-LSTM showed improved prediction performance for systolic and diastolic blood pressure estimation. Among all compared models, CBAM-CNN-BiLSTM achieved the best overall performance. However, the performance gaps among the architectural variants were relatively modest, indicating limited gains from architectural refinement under the fixed input condition. To evaluate the impact of architectural modifications, Table 8 summarizes the performance gains in ME, MAE, and STD for three model variants—CNN-BiLSTM, CBAM-CNN-LSTM, and CBAM-CNN-BiLSTM—relative to the baseline CNN-LSTM model. Positive gain values indicate improved estimation accuracy for systolic and diastolic blood pressure compared to the baseline, while negative values denote performance degradation. As shown, CBAM-CNN-BiLSTM achieved the highest overall gains, albeit with modest improvements over other variants.

### 3.3. Comparison with Related Works

Table 9 summarizes the comparison between the best-performing configuration identified in this study and previous blood pressure estimation studies based on the UCI dataset. Among the compared studies, only the present study and Wang et al. [6] achieved relatively better performance in terms of estimation accuracy.

Compared with Wang et al. [12] (Study V), the selected model in this study achieved lower error metrics for both systolic and diastolic blood pressure estimation. These findings indicate that the model configuration selected through the present comparative analysis is highly competitive on the UCI dataset.

### 3.4. Real-World Validation

To further examine the practical feasibility of the best-performing configuration identified in this study, the final model was deployed on an Orange Pi Zero2 platform integrated with a wearable PPG optical sensor. A total of 26 participants were enrolled in the real-world validation experiment, including 8 normotensive, 8 prehypertensive, and 10 hypertensive individuals. All participants provided written informed consent. Reference blood pressure values were obtained using an Omron 7206 cuff-based oscillometric monitor, while synchronized PPG signals were collected using the on-board wearable sensor for real-time BP estimation.

**Figure 7:**
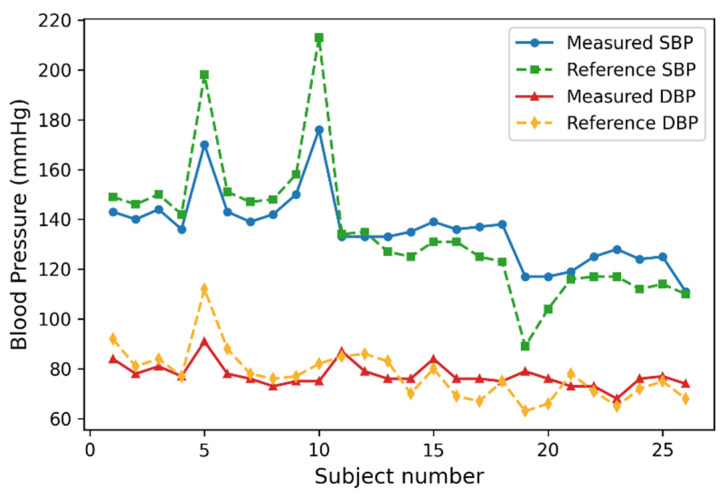
Differences between reference and measured values.

The estimated SBP and DBP values were compared with the corresponding Omron reference measurements, as shown in Figure 7. Overall, the predicted values showed similar subject-wise trends to the reference measurements across the 26 participants. For both SBP and DBP, the model outputs generally varied in accordance with the reference BP levels among normotensive, prehypertensive, and hypertensive participants, although noticeable deviations were observed in some individual cases.

**Figure 8:**
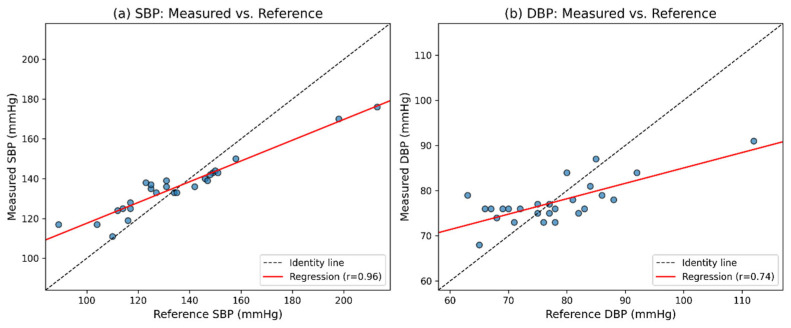
Scatter plots of estimated versus reference blood pressure values for SBP (**a**) and DBP (**b**).

**Figure 9:**
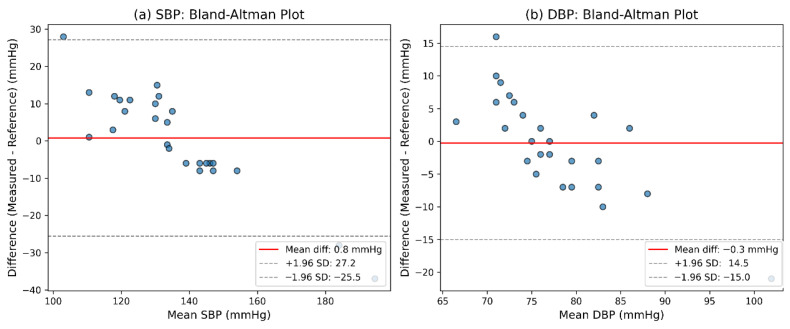
Bland–Altman plots of estimated and reference blood pressure values for SBP (**a**) and DBP (**b**).

Scatter plots and Bland–Altman analyses of the real-world validation results are presented in Figure 8 and Figure 9, respectively. The numerical statistical results, including error-based metrics, correlation coefficients, and Bland–Altman summary statistics, are summarized in Table 10. In addition, the detailed measurement results for all 26 participants, including the model-predicted SBP/DBP values and the corresponding Omron-recorded reference values, are provided in Appendix A Table A1.

## 4. Discussion

### 4.1. Effect of Signal Representation

**Figure 10:**
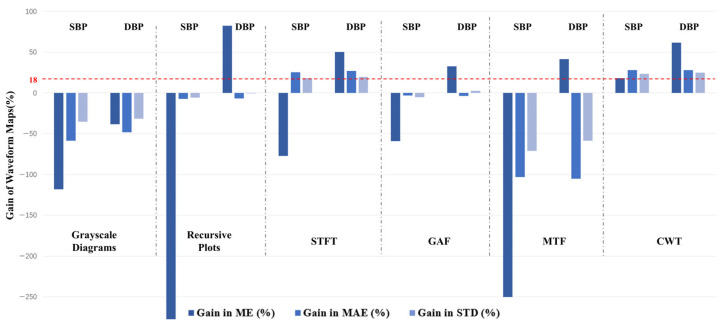
Percentage gains in ME, MAE, and STD for different signal representation methods relative to the baseline Waveform Maps.

This study evaluated seven commonly used PPG signal representation methods for blood pressure prediction. As shown in Table 5, STFT and CWT achieved the most competitive performance. In particular, CWT achieved the best overall performance among the seven evaluated representations. These findings suggest that blood-pressure-related information in PPG is not sufficiently characterized by waveform shape alone, but is also associated with dynamic patterns distributed across time and frequency. Figure 10 further shows the relative gains of the six transformed representations over the baseline waveform maps, indicating that different signal processing methods preserve task-relevant information to different extents.

The better performance of CWT can be understood from the perspective of information preservation in non-stationary physiological signals. PPG is a time-varying peripheral hemodynamic signal, and its relevant blood-pressure-related patterns may appear at different temporal scales and in localized waveform variations. Compared with methods that mainly retain global morphology or impose a fixed transformation structure, CWT provides a multi-scale time–frequency representation that is more capable of preserving localized and scale-dependent signal characteristics. This may explain why it outperformed the other evaluated methods in this study. At the same time, some alternative representations showed limited or even negative gains in certain metrics, suggesting that inappropriate signal processing may weaken, distort, or discard useful information and thereby lead to inferior prediction performance. Therefore, signal representation is not merely a preprocessing step, but a key factor that constrains the amount of blood-pressure-relevant information available to the model.

From a physiological perspective, the present results support the view that informative PPG characteristics are likely distributed across both waveform morphology and dynamic temporal variation, rather than being confined to a single point or simple descriptor. However, our study was designed to compare representation methods rather than to identify specific physiological features or quantify their individual contributions. Therefore, the present results do not allow us to conclude which exact waveform components are the most decisive, nor do they prove that CWT is the universally optimal representation for cuffless blood pressure estimation. Instead, our findings indicate that, among the seven methods evaluated here, the representations that better preserve multi-scale and time-varying characteristics—especially CWT and STFT—are more effective for this prediction task. This also implies that other signal representations not investigated in this study may potentially achieve even better performance.

### 4.2. Effect of Architectural Refinement

**Figure 11:**
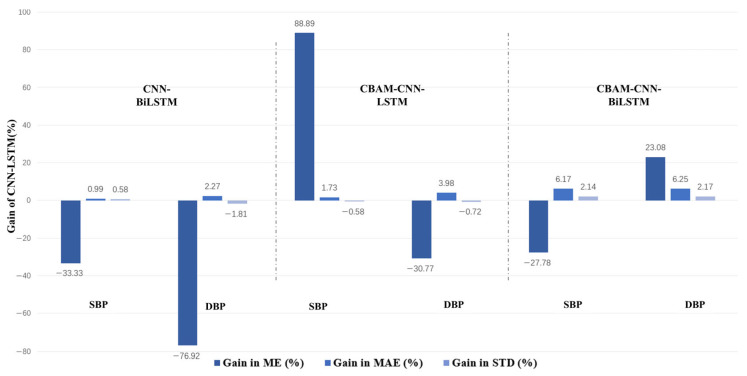
Percentage gains in ME, MAE, and STD for different model variants relative to the baseline CNN-LSTM model.

As shown in Table 7, the improvements brought by architectural refinement were relatively modest compared with those obtained from signal transformation. Although both CBAM and BiLSTM improved the estimation performance over the baseline CNN-LSTM model, the differences among architectural variants remained limited. This suggests that, once a reasonably informative signal representation has been established, the additional gains obtained from increasing architectural complexity may be comparatively small. In other words, for PPG-based cuffless blood pressure estimation, the choice of input representation appears to have a greater impact on performance than model refinement alone. Figure 11 further shows the relative gains of the three architectural variants over the baseline CNN-LSTM model, indicating that incorporating bidirectional recurrent structures and attention mechanisms improves blood pressure estimation performance to varying extents. Among them, the CBAM-CNN-BiLSTM model achieves the highest overall gains, albeit with modest improvements over other variants.

This result can be interpreted from the role of architecture in the learning process. Architectural refinement mainly affects how effectively the network extracts and integrates information already contained in the input representation. For example, CBAM may improve feature selection by emphasizing more informative channels and local regions, while BiLSTM may improve temporal modeling by incorporating bidirectional context. However, these refinements cannot fully compensate for useful physiological information that has already been weakened or lost during preprocessing. Therefore, the relatively limited gains from architecture refinement further support the conclusion that preserving blood-pressure-relevant information at the signal representation stage is more critical than simply increasing model complexity. Future studies should therefore prioritize robust and physiologically meaningful signal representation, while treating architectural refinement as a complementary strategy.

### 4.3. Robustness Under Real-World Conditions

The real-world validation results showed lower accuracy compared to the retrospective UCI-based experiments, which can be attributed to several factors. First, the model was deployed on the hardware platform without any fine-tuning or retraining on real-world data, meaning it had to generalize directly from the retrospective training distribution to an entirely unseen hardware–participant environment. Second, unlike the UCI dataset, which underwent strict signal quality assessment and band-pass filtering, the real-world validation used raw PPG signals collected from a wearable sensor without such quality screening. Consequently, the real-world data contained various noise sources, including motion artifacts, baseline drift, and contact-pressure variations, which degraded the effective signal-to-noise ratio and increased estimation errors. Third, the reference labels in the real-world validation were obtained using an Omron cuff-based oscillometric monitor, whereas the UCI dataset used invasive arterial blood pressure (ABP) as the ground truth. Cuff-based oscillometric devices typically have inherent measurement errors of 3–5 mmHg compared with intra-arterial pressure, and their readings can be further affected by cuff placement, posture, and physiological variability. Therefore, the reference labels themselves carry uncertainty that contributes to the observed discrepancy between predicted and reference values.

Given these factors, the real-world results should be interpreted with caution. The performance decline does not necessarily indicate a failure of the proposed methodology, but rather reflects the challenges of translating a retrospectively optimized model to real-world conditions without adaptation. Importantly, despite these challenges, the model still achieved a strong correlation with the Omron reference measurements (R = 0.96 for SBP and R = 0.74 for DBP), demonstrating good consistency between the proposed method and a widely used cuff-based device.

In this context, the primary role of the real-world validation is to assess preliminary practical consistency and correlation, rather than to establish absolute clinical performance. More broadly, this study should be regarded as a comparative methodological investigation of signal representation and model design for cuffless BP estimation, with the real-world results providing supportive evidence of practical feasibility under controlled interpretation.

### 4.4. Limitations and Future Work

Although this study has achieved encouraging results, several limitations should be acknowledged. First, constrained by ethical considerations and the limited number of hypertensive patients in real-world settings, the validation cohort remains relatively small, which limits the generalizability of the findings. Second, the retrospective dataset used in this study was processed according to relatively strict criteria regarding signal duration, reference BP values, missing data, and signal quality. These criteria were adopted with reference to previously published studies to reduce obvious interference and to provide a standardized experimental setting for fair comparison among different signal representation methods and model architectures. However, this conservative preprocessing strategy may have reduced the physiological and clinical heterogeneity of the analyzed cohort. Third, the current study adopted a segment-level partitioning strategy rather than a subject-independent protocol. As a result, segments from the same subject may appear in both the training and test sets, and subject-specific characteristics cannot be fully excluded. Therefore, the reported absolute performance may be optimistically biased and should be interpreted primarily as relative comparisons among methods, rather than as definitive evidence of generalization to unseen individuals in real-world deployment scenarios.

Future studies should include larger and more diverse external cohorts, adopt subject-level partitioning, and further evaluate the robustness and generalizability of the proposed framework under real-world conditions, with particular attention to severe hypertension and comorbid cardiovascular diseases such as atrial fibrillation.

## 5. Conclusions

This study systematically compared different signal transformation methods and architectural variants for PPG-based cuffless blood pressure estimation. The results showed that signal representation had a greater impact on estimation performance than architectural refinement, with CWT achieving the best overall performance among the evaluated transformation methods. These findings emphasize the importance of appropriate signal representation in cuffless blood pressure estimation and provide a basis for the future development of wearable sensor-based cuffless blood pressure monitoring systems.

## Figures and Tables

**Table 1:** Summary of preprocessing steps and corresponding data output.

Preprocessing Step	Criterion/Method	Output Data
Subject selection	Records with duration ≥ 500 s	1741 subjects
Signal segmentation	5 s segmentation	191,598 segments
Reference BP filtering	Removal of segments with invalid reference BP values	190,114 segments
PPG quality assessment	Skewness > 0.1 and kurtosis < −0.1	171,514 segments
Band-pass filtering	Butterworth band-pass filter (0.5–10 Hz)	171,514 segments
Normalization	Mean-variance normalization	171,514 segments

**Table 2:** Comparison of commonly used signal transformation methods for PPG representation.

**Full Name**	**Formula**	**Description**	**Ref.**
**Waveform maps**	x(t) vs. t	Plot of signal amplitude over time.	[8]
**Grayscale** **diagrams**	$G(i)=\mathrm{round}(255\cdot \frac{x_{i}-x_{min}}{x_{max}-x_{min}})$	Conversion of time series into grayscale intensity values.	[22]
**Recurrence** **plots**	$R_{i,j}=\Theta (\varepsilon -∥x_{i}-x_{j}∥)$	Binary matrix indicating state recurrences in phase space.	[23]
**Short-time Fourier transform (STFT)**	$\mathrm{STFT}(\tau ,\omega )=\int x(t)w(t-\tau )e^{-j\omega t}dt$	Time–frequency representation using a sliding window.	[24]
**Gramian angular field (GAF)**	$cos(\phi_{i}+\phi_{j})$ or $cos(\phi_{i}-\phi_{j})$	Encoding of time series as polar coordinates in a Gram matrix.	[25]
**Markov transition field (MTF)**	$M_{i,j}=P(x_{t+1}=q_{j}|x_{t}=q_{i})$	Matrix of Markov transition probabilities between quantized states.	[26]
**Continuous wavelet transform (CWT)**	$\mathrm{CWT}(a,b)=\int x(t)\psi_{a,b}^{*}(t)dt$	Multi-scale decomposition via wavelet basis functions.	[27]

**Table 3:** Layer-Wise Architecture of the CNN-LSTM Network.

Layer	Type	Kernel Size	Stride	Kernel	Input Size
1	Conv2D	3 × 3	1	32	128 × 128 × 3
2	Conv2D	3 × 3	1	32	128 × 128 × 32
3	MaxPooling2D	2 × 2	2	-	128 × 128 × 32
4	Conv2D	3 × 3	1	64	64 × 64 × 32
5	MaxPooling2D	2 × 2	2	-	64 × 64 × 64
6	Conv2D	3 × 3	1	96	32 × 32 × 64
7	MaxPooling2D	2 × 2	2	-	32 × 32 × 96
8	Conv2D	3 × 3	1	128	16 × 16 × 96
9	MaxPooling2D	2 × 2	2	-	16 × 16 × 128
10	Conv2D	3 × 3	1	160	8 × 8 × 128
11	MaxPooling2D	2 × 2	2	-	8 × 8 × 160
12	Conv2D	3 × 3	1	192	4 × 4 × 160
13	MaxPooling2D	2 × 2	2	-	4 × 4 × 192
14	LSTM	-	-	-	4 × 192
15	LSTM	-	-	-	4 × 192
16	Fully connected	-	-	24	768
17	Fully connected	-	-	1	24

**Table 4:** Overview of the architectural variants.

Model	Based on CNN-LSTM	CBAM	BiLSTM
**CNN-LSTM**	√	×	×
**CNN-BiLSTM**	√	×	√
**CBAM-CNN-LSTM**	√	√	×
**CBAM-CNN-BiLSTM**	√	√	√

**Table 5:** Comparative performance of seven signal representation methods under the CNN-LSTM framework.

Method	Target	ME (mmHg)	MAE (mmHg)	STD (mmHg)	95% CI of ME	95% LoA
Waveform Maps	SBP	−0.22	5.62	6.69	(−0.29, −0.15)	(−13.33, 12.89)
	DBP	0.34	2.44	3.66	(0.30, 0.38)	(−6.83, 7.51)
Grayscale Diagrams	SBP	−0.48	8.90	9.06	(−0.58, −0.38)	(−18.24, 17.28)
	DBP	0.47	3.62	4.82	(0.42, 0.52)	(−8.98, 9.92)
Recursive Plots	SBP	0.83	6.04	7.06	(0.75, 0.90)	(−13.01, 14.67)
	DBP	**0.06**	2.61	3.69	(0.02, 0.10)	(−7.17, 7.29)
STFT	SBP	0.39	4.20	5.47	(0.33, 0.45)	(−10.33, 11.11)
	DBP	0.17	1.78	2.94	(0.14, 0.20)	(−5.59, 5.93)
GAF	SBP	0.35	5.80	7.03	(0.28, 0.42)	(−13.43, 14.13)
	DBP	0.23	2.53	3.57	(0.19, 0.27)	(−6.77, 7.23)
MTF	SBP	0.77	11.40	11.43	(0.65, 0.89)	(−21.63, 23.17)
	DBP	0.20	5.01	5.80	(0.14, 0.26)	(−11.17, 11.57)
CWT	SBP	**0.18**	**4.05**	**5.13**	(0.13, 0.23)	(−9.87, 10.23)
	DBP	0.13	**1.76**	**2.76**	(0.10, 0.16)	(−5.28, 5.54)

Note: SBP is Systolic Blood Pressure; DBP is Diastolic Blood Pressure. Bold text indicates the optimal result for each indicator.

**Table 6:** Performance gain of other methods relative to the baseline Waveform Maps under the CNN-LSTM framework.

Models	Target	Δ|ME| (mmHg)	Gain in ME (%)	Δ|MAE| (mmHg)	Gain in MAE (%)	Δ|STD| (mmHg)	Gain in STD (%)
Grayscale Diagrams	SBP	−0.26	−118.18	−3.28	−58.36	−2.37	−35.43
	DBP	−0.13	−38.24	−1.18	−48.36	−1.16	−31.69
Recursive Plots	SBP	−0.61	−277.27	−0.42	−7.47	−0.37	−5.53
	DBP	0.28	82.35	−0.17	−6.97	−0.03	−0.82
STFT	SBP	−0.17	−77.27	1.42	25.27	1.22	18.24
	DBP	0.17	50	0.66	27.05	0.72	19.67
GAF	SBP	−0.13	−59.09	−0.18	−3.2	−0.34	−5.08
	DBP	0.11	32.35	−0.09	−3.69	0.09	2.46
MTF	SBP	−0.55	−250	−5.78	−102.85	−4.74	−70.85
	DBP	0.14	41.18	−2.57	−105.33	−2.14	−58.47
CWT	SBP	0.04	18.18	1.57	27.94	1.56	23.32
	DBP	0.21	61.76	0.68	27.87	0.9	24.59

Note: SBP is Systolic Blood Pressure; DBP is Diastolic Blood Pressure.

**Table 7:** Prediction performance of datasets processed with CWT on different CNN-LSTM models.

Models	Target	ME (mmHg)	MAE (mmHg)	STD (mmHg)	95% CI of ME (mmHg)	95% LoA (mmHg)
CNN -LSTM	SBP	0.18	4.05	5.13	(0.13, 0.23)	(−9.87, 10.23)
	DBP	0.13	1.76	2.76	(0.10, 0.16)	(−5.28, 5.54)
CNN -BiLSTM	SBP	0.24	4.01	5.10	(0.19, 0.29)	(−9.76, 10.24)
	DBP	0.23	1.72	2.81	(0.20, 0.26)	(−5.28, 5.74)
CBAM-CNN -LSTM	SBP	**0.02**	3.98	5.16	(−0.03, 0.08)	(−10.09, 10.13)
	DBP	0.17	1.69	2.78	(0.14, 0.20)	(−5.28, 5.62)
CBAM-CNN -BiLSTM	SBP	−0.23	**3.80**	**5.02**	(−0.28, −0.18)	(−10.07, 9.61)
	DBP	**0.10**	**1.65**	**2.70**	(0.07, 0.13)	(−5.19, 5.39)

Note: SBP is Systolic Blood Pressure; DBP is Diastolic Blood Pressure. Bold text indicates the optimal result for each indicator.

**Table 8:** Performance gain (%) of architectural variants relative to the baseline CNN-LSTM model.

Models	Target	Δ|ME| (mmHg)	Gain in ME (%)	Δ|MAE| (mmHg)	Gain in MAE (%)	Δ|STD| (mmHg)	Gain in STD (%)
CNN-BiLSTM	SBP	−0.06	−33.33	0.04	0.99	0.03	0.58
	DBP	−0.1	−76.92	0.04	2.27	−0.05	−1.81
CBAM-CNN-LSTM	SBP	0.16	88.89	0.07	1.73	−0.03	−0.58
	DBP	−0.04	−30.77	0.07	3.98	−0.02	−0.72
CBAM-CNN-BiLSTM	SBP	−0.05	−27.78	0.25	6.17	0.11	2.14
	DBP	0.03	23.08	0.11	6.25	0.06	2.17

Note: SBP is Systolic Blood Pressure; DBP is Diastolic Blood Pressure.

**Table 9:** Comparison of studies related to blood pressure prediction using the UCI dataset.

Study	Pre-Processing	Models	Target	ME (mmHg)	MAE (mmHg)	STD (mmHg)
Khan et al. [29]	PPG and ECG feature extraction	Penalized regression	SBP	-	5.01	-
			DBP	-	4.27	-
Rong et al. [30]	Time–frequency and waveform plots	Multimodal fusion Models	SBP	−1.13	5.59	7.25
			DBP	0.14	3.36	4.48
Wang et al. [31]	PPG feature extraction	LASSO-LSTM	SBP	-	4.19	-
			DBP	-	1.71	-
Wang et al. [32]	PPG to VG chart	CNN	SBP	−0.08	7.42	10.10
			DBP	0.06	3.40	4.80
Wang et al. [12]	PPG to VG chart and GAF conversion	LeNet-5	SBP	0.18	4.67	7.46
			DBP	0.34	2.48	4.07
Wang et al. [6]	PPG to VG chart	Two-way input AlexNet	SBP	**0.01**	6.17	8.46
			DBP	**−0.04**	3.66	5.36
This study	PPG conversion to CWT	CBAM-CNN -BiLSTM	SBP	−0.23	**3.80**	**5.02**
			DBP	0.10	**1.65**	**2.70**

Note: SBP is Systolic Blood Pressure; DBP is Diastolic Blood Pressure. Bold text indicates the optimal result for each indicator.

**Table 10:** Prediction performance on the external validation set.

Target	ME (mmHg)	MAE (mmHg)	STD (mmHg)	95% CI of ME (mmHg)	R
SBP	0.42	9.42	13.57	(−4.80, 5.65)	0.96
DBP	0.81	5.77	6.78	(−1.80, 3.41)	0.74

Note: Reference values in this external validation were obtained using an Omron cuff-based oscillometric monitor, not invasive arterial blood pressure. Results are for reference purposes only.

## Data Availability

Data are contained within the article.

## Appendix A

**Table A1:** Detailed Measurement Results for Each Subject.

Grade	Subject No.	Measured SBP	Reference SBP	Measured DBP	Reference DBP
**HT**	1	143	149	84	92
	2	140	146	78	81
	3	144	150	81	84
	4	136	142	77	77
	5	170	198	91	112
	6	143	151	78	88
	7	139	147	76	78
	8	142	148	73	76
	9	150	158	75	77
	10	176	213	75	82
**PHT**	11	133	134	87	85
	12	133	135	79	86
	13	133	127	76	83
	14	135	125	76	70
	15	139	131	84	80
	16	136	131	76	69
	17	137	125	76	67
	18	138	123	75	75
**NT**	19	117	89	79	63
	20	117	104	76	66
	21	119	116	73	78
	22	125	117	73	71
	23	128	117	68	65
	24	124	112	76	72
	25	125	114	77	75
	26	111	110	74	68
